# Longitudinal analysis of the behavioral phenotype in a novel transgenic rat model of early stages of Alzheimer's disease

**DOI:** 10.3389/fnbeh.2014.00321

**Published:** 2014-09-16

**Authors:** Pablo Galeano, Pamela V. Martino Adami, Sonia Do Carmo, Eduardo Blanco, Cecilia Rotondaro, Francisco Capani, Eduardo M. Castaño, A. Claudio Cuello, Laura Morelli

**Affiliations:** ^1^Laboratorio de Amiloidosis y Neurodegeneración, Instituto de Investigaciones Bioquímicas de Buenos Aires, Fundación Instituto Leloir, CONICETCiudad Autónoma de Buenos Aires, Argentina; ^2^Instituto de Investigaciones Cardiológicas “Prof. Dr. Alberto C. Taquini” (ININCA), Universidad de Buenos Aires and CONICETCiudad Autónoma de Buenos Aires, Argentina; ^3^Department of Pharmacology and Therapeutics, McGill UniversityMontreal, QC, Canada; ^4^Departament de Pedagogia i Psicologia, Facultatd'Educació, Psicologia i Treball Social, Universitat de LleidaLleida, Spain

**Keywords:** Alzheimer's disease, cognitive impairment, anxiety, amyloid β, transgenic rat models

## Abstract

Intraneuronal accumulation of amyloid β (iAβ) has been linked to mild cognitive impairment that may precede Alzheimer's disease (AD) onset. This neuropathological trait was recently mimicked in a novel animal model of AD, the hemizygous transgenic McGill-R-Thy1-APP (Tg^+/−^) rat. The characterization of the behavioral phenotypes in this animal model could provide a baseline of efficacy for earlier therapeutic interventions. The aim of the present study was to undertake a longitudinal study of Aβ accumulation and a comprehensive behavioral evaluation of this transgenic rat model. We assessed exploratory activity, anxiety-related behaviors, recognition memory, working memory, spatial learning and reference memory at 3, 6, and 12 months of age. In parallel, we measured Aβ by ELISA, Western blots and semiquantitative immunohistochemistry in hippocampal samples. SDS-soluble Aβ peptide accumulated at low levels (~9 pg/mg) without differences among ages. However, Western blots showed SDS-resistant Aβ oligomers (~30 kDa) at 6 and 12 months, but not at 3 months. When compared to wild-type (WT), male Tg^+/−^ rats exhibited a spatial reference memory deficit in the Morris Water Maze (MWM) as early as 3 months of age, which persisted at 6 and 12 months. In addition, Tg^+/−^ rats displayed a working memory impairment in the Y-maze and higher anxiety levels in the Open Field (OF) at 6 and 12 months of age, but not at 3 months. Exploratory activity in the OF was similar to that of WT at all-time points. Spatial learning in the MWM and the recognition memory, as assessed by the Novel Object Recognition Test, were unimpaired at any time point. The data from the present study demonstrate that the hemizygous transgenic McGill-R-Thy1-APP rat has a wide array of behavioral and cognitive impairments from young adulthood to middle-age. The low Aβ burden and early emotional and cognitive deficits in this transgenic rat model supports its potential use for drug discovery purposes in early AD.

## Introduction

Alzheimer's disease (AD) is a neurodegenerative disorder that frequently begins with memory complaints progressing to a severe deterioration of multiple memory systems (Eustache et al., 2006). In addition to cognitive impairment, AD patients exhibit several neuropsychiatric symptoms with the progression of the disease (Geda et al., 2013). Some of the classical neuropathological changes in AD are the presence of amyloid β (Aβ) deposits and neurofibrillary tangles which initiate in the entorhinal cortex and hippocampus, and spread to the medial temporal lobe (Scahill et al., 2002). This progression of neuropathology leads to episodic and semantic memory deficits that can be detected at early stages of the disease (Bondi et al., 1999, 2008; Collie and Maruff, 2000; Schmitt et al., 2000; Smith et al., 2007). As the disease progresses, other cognitive domains, such as attention and executive function, among others, are affected (Bondi et al., 2008).

Recent reports suggest that intraneuronal accumulation of Aβ (iAβ) takes place with the aging of the human brain many years prior to Aβ plaques accumulation and Alzheimer's disease onset (Gouras et al., 2000; D'Andrea et al., 2001). Aging is the predominant risk factor for AD and there is biochemical evidence supporting that the disease has an extensive preclinical phase (Sperling et al., 2011; Bateman et al., 2012). In this context, preventive medicine combined with early diagnosis may allow a substantial proportion of aged people to escape from suffering AD. The relevance of the early stage of AD is further supported by the failure of recent clinical trials aimed at reducing extracellular Aβ levels and plaque formation (Laurijssens et al., 2013; Vellas et al., 2013). Some of the reasons proposed for the failure may be the initiation of the trials in demented patients with serious brain damage together with unforeseen design flaws in the studies based on results from inadequate animal models (Laurijssens et al., 2013; Vellas et al., 2013).

To test potential novel drug therapies specific for early stages of AD it is necessary to develop an animal model at a pre-plaque stage with a progressive decline in emotional and cognitive function. The phenotype should be sensitive to iAβ accumulation and aging. Transgenic mouse models of AD have been widely used to model the impairments in some cognitive domains shown in patients with AD, such as semantic, working and recognition memory (Webster et al., 2014). Semantic memory refers to a conscious recollection and retrieval of factual information and in rodents is most closely correlated with reference memory. The latter implies the learning of certain aspects of a task that remain constant throughout the behavioral test (Webster et al., 2014). Working memory is a system that holds information for a limited amount of time, allowing its use and manipulation to guide behaviors (Baddeley, 2003). Recognition memory belongs to the long-term declarative memory system and consists in the ability to recognize individuals, events and objects that were already encountered (Ally, 2012).

Available transgenic mice of AD have a general difficulty in recreating the characteristics of early AD as most of them show an aggressive neuropathological phenotype characterized by a massive plaque formation (Do Carmo and Cuello, 2013). Transgenesis in rats offers great potential to decipher subtle and early aspects of AD pathology. The rat is, in many aspects, closer to humans than mice. Because of its complex CNS and its predictable and multi-faceted behavioral display, this species is of great value for accurate cognitive assessment (Whishaw et al., 2001; Abbott, 2004). Different transgenic rat models of AD have been developed in the last 10 years (Do Carmo and Cuello, 2013) however, only two models, the UKUR25 (Echeverria et al., 2004) and the hemizygous McGill-R-Thy1-APP (Leon et al., 2010) show exclusively iAβ accumulation in cortex and hippocampus without amyloid plaques or tangles at later stages. The difference between both models is that McGill-R-Thy1-APP rats show progressive amyloid pathology from postnatal week one while UKUR25 rats from 6 months of age (Echeverria et al., 2004; Leon et al., 2010). In contrast to other transgenic rat models of AD, such as UKUR25 and TgF344-AD in which iAβ accumulation is sufficient to increase p-Tau levels, available bibliographic reports do not indicate that McGill-R-Thy1-APP rat model develops Tau pathology (Do Carmo and Cuello, 2013). This discrepancy can be explained by differences in the genetic background and the transgene expressed.

The McGill-R-Thy1-APP model was previously evaluated in the Morris Water Maze (MWM) test (Leon et al., 2010). MWM is the most widely employed spatial learning and memory task systematically impaired in all of the different strains of AD transgenic mice (Webster et al., 2014). Recently, it was also reported that the hemizygous transgenic McGill-R-Thy1-APP (Tg^+/−^) rat, the least genetically aggressive AD transgenic model developed so far, also showed impairments in auditory fear conditioning and in the Novel Object Recognition and Location (NORL) task as early as 3 months of age (Iulita et al., 2014). However, it was not indicated if by this age Tg^+/−^ rats accumulate soluble Aβ peptides as compared to wild-type littermates (Iulita et al., 2014). In addition, working memory and the temporal progression of AD-like non-cognitive behavioral abnormalities, which are affected early during the course of AD, were not determined in this novel transgenic animal model.

Multiple behavioral tasks should ideally be used to characterize the phenotypes of potential animal models of AD since in the clinical setting, a wide range of neuropsychological tests are employed to determine the profile of the cognitive impairment (Gainotti et al., 2014; Webster et al., 2014). In the present study, by using a longitudinal assessment procedure, we sought to determine the levels and accumulation of soluble Aβ and iAβ peptides and the development of impairments in the exploratory activity, emotional behavior and working and spatial reference memory in Tg^+/−^ rats. In this way, we will test if this model recapitulates some of the biochemical, emotional and cognitive alterations seen in pre-symptomatic AD. Our results could be relevant to define in this animal model the critical window for therapeutic interventions that may be applied to early stages of AD.

## Materials and methods

### Ethical statements

The study was carried out in strict accordance with the guidelines of the OLAW-NIH (Office Laboratory Animal Welfare). The protocol was approved by the local Animal Care Committee of Fundación Instituto Leloir (FIL) Assurance# A5168-01.

### Animals

Hemizygous transgenic McGill-R-Thy1-APP (Tg^+/−^) rats, harboring the human APP751 transgene with the Swedish and Indiana mutation under the control of the murine Thy1.2 promoter (Leon et al., 2010), were provided to Fundación Instituto Leloir (FIL) by The Royal Institution for the Advancement of Learning/McGill University, Montreal, Quebec (Canada).

### Husbandry and genotyping

An in-house breeding colony was established at FIL by cross-breeding Tg^+/−^ males with wild-type Wistar females rats. Each litter was culled to 10 pups (up to 8 males per litter were left). At weaning (21 post-natal days), dams were removed from the litters and pups were genotyped as described below. When the results of the genotyping were available (between 22 and 23 post-natal days), rats were pseudorandomly assigned to experimental groups. To avoid the litter effect, groups were made up of pups from 3 to 4 different litters. Animals were maintained on a 12-h light/12-h dark cycle, group-housed 2–4 per cage and had *ad libitum* access to water and a standard rodent diet. Tissue samples were obtained by ear punches from the litters to determine the genotype of each rat. Genotyping was performed using a validated protocol. Briefly, genomic DNA was prepared from ear biopsy tissue using proteinase K digestion, followed by phenol/chloroform/isoamyl alcohol extraction (Life Technologies). DNA quantification was determined in a Nanodrop 2000 spectrofotometer. Samples with a ratio of absorbance at 260/280 and 260/230 nm above 1.80 were used for PCR to recognize whether or not the rat carried the human APP (hAPP) gene. The following sets of primers were used: APP-F 5′-AGGACTGACCACTCGACCAG-3′; APP-R 5′-CGGGGGTCTAGTTCTGGAT-3′; CyclophilinG-F 5′- TACAACAGTAGAACAAGGGAGCGAAG-3′ and CyclophilinG-R 5′-ATCCCTCCTTCTTCTCCTCCTATCTTT-3′. Cycling conditions were: 95°C (15 min) and 40 cycles of 94°C (30 s), 54°C (1 min), 72°C (1 min) followed by one step at 72°C (2 min) and kept at 4°C until processed. PCR products were resolved by 2% agarose mini-gel electrophoresis in TBE buffer and visualized with ethidium bromide (10 mg/ml) staining under UV light. Cyclophilin G amplicon (248 bp) was used as a housekeeping gene to verify DNA integrity and PCR conditions. The hAPP amplicon (387 bp) indicates the presence of the human transgene. To verify the hemizygocity of the transgenic rats, quantitative PCR (qPCR) was performed by using the same set of hAPP primers as described above and the following set of GAPDH (used as housekeeping gene) primers: GAPDH-F: 5′-GGGGAAGGACGCTGTACGGG-3′ and GAPDH-R: 5′-AAGGGGAGC AACAGCTGGGGT-3′. A total of 1 ng of genomic DNA was used per well (5 μl of a 0.2 ng/μl dilution). Samples were plated in triplicate. SYBR-Green qPCR was performed by using the KAPA SYBR^®^ FAST Universal 2X qPCR Master Mix (Kapa Biosystems). Reactions were run in a Stratagene Mx3005P cycler and results analyzed by the MxPro software in a Comparative Quantitation mode. In all of the runs a genomic DNA sample of a hemizygous animal was included and used as an internal calibrator. If the unknown sample is hemizygous, the relative quantity (dRn) value should be between 0.8 and 1.3; if the unknown sample is homozygous the result is close to a value of 2 (between 1.8 and 2.3).

### Experimental design

For time-course analysis of the behavioral phenotype, 3 cohorts of male rats consisting in Tg^+/−^ and wild-type (WT) littermates were used. Rats were divided in 6 experimental groups as follows: group 1 (3 months old Tg^+/−^ rats; *n* = 10); group 2 (3 months old WT rats; *n* = 9); group 3 (6 months old Tg^+/−^ rats; *n* = 9); group 4 (6 months old WT rats; *n* = 7); group 5 (12 months old Tg^+/−^ rats; *n* = 11); group 6 (6 months old WT rats; *n* = 11) (see **Figure 2**). Only male rats were used to avoid any potential effects of changes in the female estrus cycle on behavioral performance.

### Hippocampal isolation

Three groups (*n* = 3/group) of naïve Tg^+/−^ male rats of 3, 6, and 12 months of age, and one group (*n* = 3) of naïve WT male rats of 12 months of age, were anesthetized with ketamine (50 mg/kg) and xylacine (10 mg/kg), perfused transaortically with 0.9% sodium chloride with 200 U/l heparine and brains were quickly removed as described previously (Leal et al., 2006). Brains were divided into left and right hemispheres. Hippocampus was isolated from left hemisphere and retained for biochemical studies. The right hemisphere was post-fixed in 4% paraformaldehyde in 0.1 M phosphate buffer (PB), pH 7.4 overnight at 4°C, and finally transferred to a solution of 30% sucrose in 0.1 M PB for 5 days, or until sectioned for immunohystochemistry into 40 μm coronal sections with a freezing sledge microtome (SM 2000R, Leica) at −20°C. None of the animals assigned to immunohistochemical and biochemical experiments were submitted to behavioral testing.

### SDS-soluble Aβ peptide quantitation

To estimate the levels of hippocampal SDS-soluble Aβ peptides, 100 mg of hippocampus were homogenized with a manual Teflon glass homogenizer in 1 ml of RIPA buffer (150 mM NaCl, 0.5% sodium deoxycolate, 1% Triton X-100, 2% SDS) containing proteases inhibitors (5 mM EDTA, 5 mM EGTA, 5 μg/ml leupeptin, 10 μg/ml aprotinin, 1 μg/ml pepstatin) and phosphatases inhibitors (50 mM sodium fluoride and 5 mM sodium orthovanadate) and centrifuged at 20,000 × g at 4°C for 45 min using a Eppendorf microcentrifuge. Supernatants were aliquoted and stored at −80°C. Individual samples were analyzed to assess SDS-soluble human Aβ40 or Aβ42 levels by using sandwich ELISA tests commercially available (Invitrogen). Results were expressed as pg of Aβ/mg of hippocampal homogenate proteins as determined with a bicinchoninic acid assay (Pierce, Rockford, IL) and were corrected by the signal of WT protein background. Determinations were performed in duplicate samples in three independent experiments. To evaluate SDS-resistant oligomeric Aβ levels, samples (250 μg/lane) were run in SDS-Tricine 12% gels and processed as previously described (Leon et al., 2010). Immunoreactivity was detected by Western blot using an enhanced chemiluminescence detection system (ECL, Thermo Scientific) and membranes analyzed and quantitated with a STORM 840 Phosphor Imager (GE Healthcare). Group values were obtained simultaneously and normalized with respect to APP immunoreactivity.

### Estimation of the volume fraction of the hippocampus affected by iAβ deposition

Brain sections were analyzed by using systematic random sampling. Every sixth brain section (240 μm apart) was stained using a free-floating immunohistochemistry procedure as previously described (Leon et al., 2010). iAβ label was detected with McSA1 (1:4000) mouse monoclonal antibody and sections were analyzed at 40× magnification by using an Olympus BX50 microscope. Immunoreactivity was assessed with Image J software (NIH, USA) in sections between the coordinates −2.30 and −3.8 mm from bregma for dorsal hippocampus and between −4.16 and −6.04 mm from bregma for ventral hippocampus (Paxinos and Watson, 2009) following a standard protocol. Stereological estimation of the hippocampal volume affected by iAβ is described below in *Image analysis* Section.

### Neurological screening and behavioral testing

One week before starting with the behavioral battery each cohort was transported to ININCA (School of Medicine, Universidad de Buenos Aires) where a behavioral core facility is available. Throughout the study period, animals were housed in a controlled environment (21 ± 2°C, 50–60% relative humidity, 12 h light/12 h dark schedule) and all efforts were made to minimize discomfort. After acclimation, rats were handled daily (5 min/day) for three consecutive days and subjected to a basic neurological examination which consisted of a battery of neurological reflex tests: righting response after being placed on the dorsal side; eye blink (response to light touch with a small camel hair brush), ear twitch, and limb withdrawal in response to tactile stimuli (light touch with a gloved finger); orienting response to a visual stimuli (flashlight) and startle response following an auditory stimulus (a metal clicker) as previously reported (Cohen et al., 2013). Responses were scored as being present or absent. The maximum time period in which behavioral tests were carried out was between 8:00 a.m. and 5 p.m. Since there is a major circadian influence on EPM and OF performance (Verma et al., 2010), these tests were conducted between 8:00 a.m. and 12:00 p.m. Moreover, testing order of the groups was counterbalanced to avoid the confounding effect of time of the day at which animals were tested. The order of tests were as follows: Elevated Plus Maze (EPM) test, Open Field (OF) test, Novel Object Recognition Test (NORT), Y-maze spontaneous alternation (Y-Maze) test, and Morris Water Maze (MWM) test. The interval between tests was at least 24 h. After each animal was tested, the entire apparatus and objects in the NORT were cleaned with 70% ethanol to prevent a bias due to olfactory cues. All testing sessions were digitally recorded (JVC Everio GZ-HD620) and analyzed using a computerized video-tracking system (Ethovision XT, version 7, Noldus Information Technology, Wageningen, The Netherlands) or the ethological observation software JWatcher V1.0. All the experimenters were blinded to the genotype of the animals.

#### Elevated plus maze test

The EPM test assesses anxiety-like behaviors (Pellow et al., 1985). The apparatus consisted of a black melamine central square platform (11 × 11 cm) from which four black melamine arms radiate (50 × 11 cm) separated from each other by 90°. Two of the arms (“closed arms”) have a wall (40 cm in height) all around its perimeter but not in the entrance and the other two arms (“open arms”) do not have any wall but with raised edges (0.25 cm) around its perimeter. The maze was elevated one meter from the floor by five legs. Light intensity in the open arms was 80–90 lux. Each rat was placed onto the central platform facing an open arm and allowed to freely explore the maze for 5 min. An arm entry was counted when rat introduced its four paws into an arm. The following dependent variables were recorded: total distance moved, number of closed arm entries, percentage of open arm entries $\left(\frac{N^{{}^{\circ}}\;open\;arm\;entries}{Total\;entries}\;\times \;100\right)$ and percentage of time spent in open arms $\left(\frac{Time\;spent\;in\;open\;arms}{300s}\;\times \;100\right)$.

#### Open field test

The OF test was used to assess spontaneous exploratory activity and anxiety-related behaviors (Walsh and Cummins, 1976). The apparatus consisted of a black melamine square (60 × 60 cm) surrounded by walls of 40 cm in height. A central area was arbitrarily defined as a square of 30 × 30 cm and it was drawn over the image of the OF in the video-tracking system. A rat was considered to be into the central area when its four paws were on it. Light intensity in the center of the OF was 70–80 lux. Each rat was placed in the center of the maze and its behavior was analyzed for 5 min. The following dependent variables were recorded: total distance moved, number of rears, number of entries into the central area and time spent in central area.

#### Novel object recognition test

The NORT evaluates recognition memory of one previously explored object (familiar) compared with one novel object (non-familiar) (Ennaceur and Delacour, 1988; Ennaceur, 2010). NORT was performed in the same OF apparatus 24 h after the OF test. Rats were tested during two trials (sample and retention trials) separated by an interval of 1 h during which they were returned to their cages. In the sample trial (duration: 5 min), each rat was faced with two identical objects (called familiar objects) placed in a symmetrical position and the time exploring freely each object was recorded. In the retention trial (duration: 3 min), one of the two familiar objects was replaced by a novel object and the time exploring each was recorded. Sets composed of three copies of the same object were used to prevent odor cues and all combinations and location of objects were used to prevent bias due to preference for a particular object or location. Exploration time was computed when the snout pointed to the object at a distance ≤2 cm. Discrimination index (d1) and discrimination ratio (d2) scores were calculated using the following formulas: d1 = *t*_*n*_ − *t*_*f*_ and $\text{d}2=\frac{t_{n}-t_{f}}{t_{n}+t_{f}}$, where *t*_*n*_ = the amount of time rats explored the novel object and *t*_*f*_ = the amount of time rats explored the familiar object.

#### Y-maze spontaneous alternation test

The Y-maze test was used to measure spatial working memory as previously described with minor modifications (Sierksma et al., 2014). The apparatus consisted of three identical arms (45 × 12 × 35 cm) diverging at 120° angle one to the other and an equilateral triangular central area. Each animal was placed in the center of the Y-maze and was free to explore the arena for 8 min. Rats tend to explore the least recently visited arm, and thus tend to alternate visits between the three arms. For efficient alternation, rats need to use working memory, and thus, they should maintain an ongoing record of most recently visited arms, and continuously update such record (Wietrzych et al., 2005). An arm entry was scored when rat placed the four paws within that arm. The following dependent variables were registered: total number of arm entries, number of triads (sequence of three consecutive visits to different arms), and percentage of alternation. An alternation was defined as an entry into three different arms on consecutive choices. The percentage of alternation was calculated as the ratio of actual to maximum number of alternations. The maximum number of possible alternations was defined as the total number of arm entries minus 2. A low percentage of alternation is indicative of an impaired spatial working memory because the rat cannot remember which arm it has just visited, and thus shows decreased spontaneous alternation.

#### Morris water maze test

The apparatus used was previously described in detail by Galeano et al. (2011). Briefly, it consisted of a circular galvanized steel pool (180 cm in diameter and 60 cm in height), painted black, filled with water at 22 ± 1°C to a depth of 36–40 cm, and imaginarily divided into four quadrants: North (N), East (E), South (S), and West (W). A cylindrical platform (10 cm in diameter and made of transparent acrylic plastic) was placed 2 cm above (visible escape platform) or beneath the water surface (hidden escape platform) in the center of one of the quadrants. To enhance the visibility of the platform during the cued learning training, a “flag” was attached to the platform. The pool was located in the center of an experimental room with multiple extra-maze visual geometric cues hanging on the wall. Indirect illumination was provided by four spiral compact fluorescent lamp in each corner facing the walls. The following dependent variables were recorded: latency to the platform, distance swum to the platform, swimming speed, time spent in each quadrant, the number of crossings over an annulus zone of 20 cm in diameter (defined as a zone surrounding the location where the platform was located during the spatial learning training), and time spent in the outer ring of the pool (25 cm wide).

***Cued learning***. During cued learning the platform protruded 2 cm above water surface and a “flag” was attached to it (visible escape platform). The maze was surrounded by black curtains to minimize the availability of extra-maze cues. For each of the four trials conducted on each day, the platform was moved to a different quadrant and a different start location was used. If a rat had not located the platform before 120 s elapsed, it was gently guided to the platform location and was allowed to remain there for 15 s. Inter-trial interval duration was 20–30 min. Two days of cued training were conducted.

***Spatial learning and reference memory***. We used procedures described previously with some modifications (Vorhees and Williams, 2006; Galeano et al., 2011). Black curtains were removed and the spatial learning phase of the task was performed over five consecutive days with four trials per day. In each session, rats were released into the pool from one of the four starting positions (one per each quadrant) and the order of the sequence was changed pseudorandomly between days. Animals were able to escape from the water using the hidden escape platform that was kept in the same location throughout the five spatial learning sessions. A trial was finished when the animal found the escape platform or when 120 s had elapsed, whichever occurred first. If a rat failed to find the platform, the experimenter guided to it by hand. Rats remained on the platform for 15 s with an inter-trial interval of 20–30 min. Twenty four hour after the last trial of the learning phase, spatial reference memory was assessed with a probe trial in which the escape platform was removed from the pool and rats were released from a new starting position not used during the learning phase. Time spent in each quadrant, the number of annulus crossings and the time spent in the outer ring of the pool (thigmotactic swimming) was recorded. After each trial rats were dried and when the session finished they were returned to their colony room.

### Image analysis

For stereological estimations the contours of ventral and dorsal hippocampus showing immunoreactivity for iAβ was drawn in each section with Image J software (NIH, USA) tool to determine the affected area. The Cavalieri's principle (Gundersen and Jensen, 1987) was used to calculate the affected volume by using the following formula: *V* = ∑*AxNxE*, where *V* = volume (mm^3^); ∑*A* = sum of area counted (mm^2^); *N* = number of sections analyzed; *E* = thickness of each section (mm).

### Statistical analysis

All data are shown as the mean ± s.e.m. Data were analyzed by Two-Way ANOVA or by Two-Way mixed ANOVA tests followed by Tukey's HSD *post-hoc* tests for multiple comparisons, unless noted otherwise. The significance level was set at 5%. SPSS 15.0 for Windows software (Chicago, IL, USA) was used to perform all statistical analyses.

## Results

### Progression of soluble Aβ and iAβ accumulation in Tg^+/−^ rats

**Figure 1:**
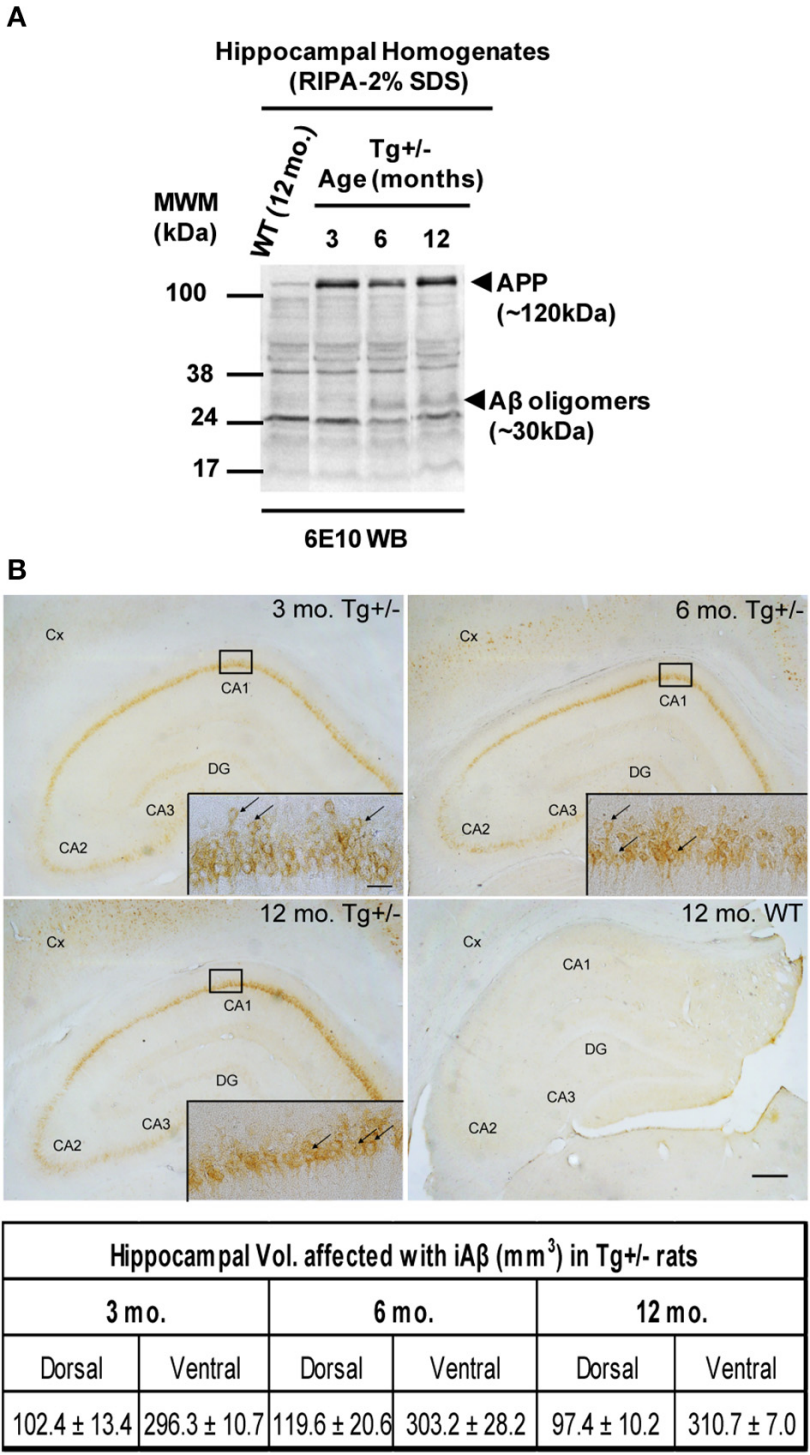
**SDS-resistant Aβ oligomers and intracellular Aβ (iAβ) accumulation in Tg^+/−^ rats. (A)** Representative Western blotting showing APP expression and Aβ oligomers by using 6E10 monoclonal antibody. Hippocampal samples of 6 and 12-month-old Tg^+/−^ rats contained Aβ oligomers (~30 kDa) in contrast to 3-month-old Tg^+/−^ or 12-month-old wild-type (WT) littermates. **(B)** iAβ is present in the hippocampus and cortex (Cx) of Tg^+/−^ animals at 3, 6, and 12 months of age (upper panels). Scale bar, 300 μm. Inset, magnification of CA1 region decorated with iAβ (arrows) in the neurons of granular layer. Scale bar, 30 μm. Hippocampal volume affected with iAβ is similar among different ages analyzed (lower panel). Three groups (*n* = 3/group) of naïve Tg^+/−^ male rats of 3, 6, and 12 months of age, and one group (*n* = 3) of naïve WT male rats of 12 months of age were assessed. These animals were not submitted to behavioral testing.

In order to better characterize Aβ-mediated neuropathology at early age, we quantified the levels of SDS-soluble and SDS-resistant peptide Aβ immunoreactivity in hippocampal homogenates from 3, 6, and 12 months old animals by a commercially available ELISA test and Western blotting, respectively. By ELISA, we detected the presence of SDS-soluble human Aβ40 in Tg^+/−^ hippocampal homogenates early at the age of 3 months (9.16 ± 1.42 pg/mg). The levels of SDS-soluble human Aβ40, however, did not differ significantly between Tg^+/−^ rats at 6 months (9.84 ± 1.30 pg/mg) or 12 months (8.00 ± 3.19 pg/mg) of age (*F* < 1), with an average of 9.00 ± 1.92 pg of Aβ40/mg of total hippocampal proteins. By contrast, we were not able to detect SDS-soluble human Aβ42 peptide in RIPA homogenates of Tg^+/−^ rats at any age analyzed. In addition, SDS-resistant Aβ oligomers appeared in hippocampal homogenates of 6 and 12 months old Tg^+/−^ rats, but not in 3 months old Tg^+/−^ rats (see Figure 1A). In accordance with previous reports, our data show that in Tg^+/−^ rats the neuropathology was restricted to iAβ (see Figure 1B, upper panel) and no significant differences were obtained in the volume fraction affected in dorsal or ventral hippocampus among different ages analyzed (*F* < 1 for both areas analyzed; see Figure 1B, lower panel). Our data strongly suggest a subtle phenotype in Tg^+/−^ rats with an “age-independent” accumulation for SDS-soluble monomeric Aβ40 and iAβ but an “age-dependent” accumulation for SDS-resistant Aβ oligomers.

**Figure 2:**
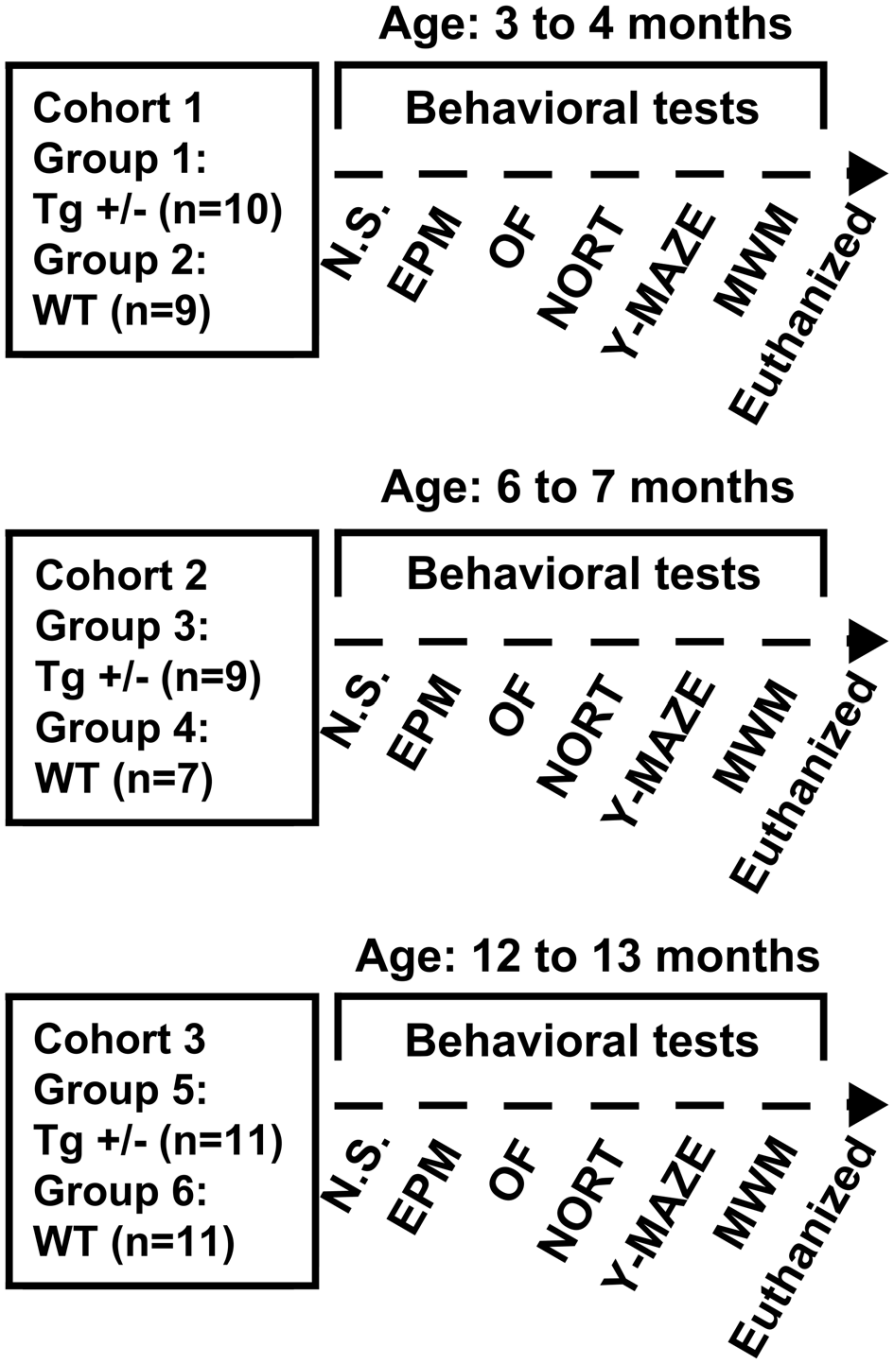
**Experimental design, cohorts' characteristics and behavioral tests employed**. Three cohorts (1, 2, and 3) of 3, 6, and 12 months old hemizygous transgenic McGill-R-Thy1-APP (Tg^+/−^) rats and their wild-type (WT) littermates were assessed in a behavioral test battery including: Elevated Plus Maze (EPM) test, Open Field (OF) test, Novel Object Recognition Test (NORT), spontaneous alternation in the Y-maze test (Y-maze), and cued and spatial reference memory versions of the Morris Water Maze (MWM) test. Prior to behavioral assessment, a neurological screening (N.S.) was performed and animals were euthanized after completion of behavioral testing.

### General health and neurological screening

Gross inspection of each rat prior to the behavioral experiments did not reveal any visible differences between WT and Tg^+/−^ animals. All animals appeared healthy and neurological reflexes were all scored as being present.

### Elevated plus maze test

**Figure 3:**
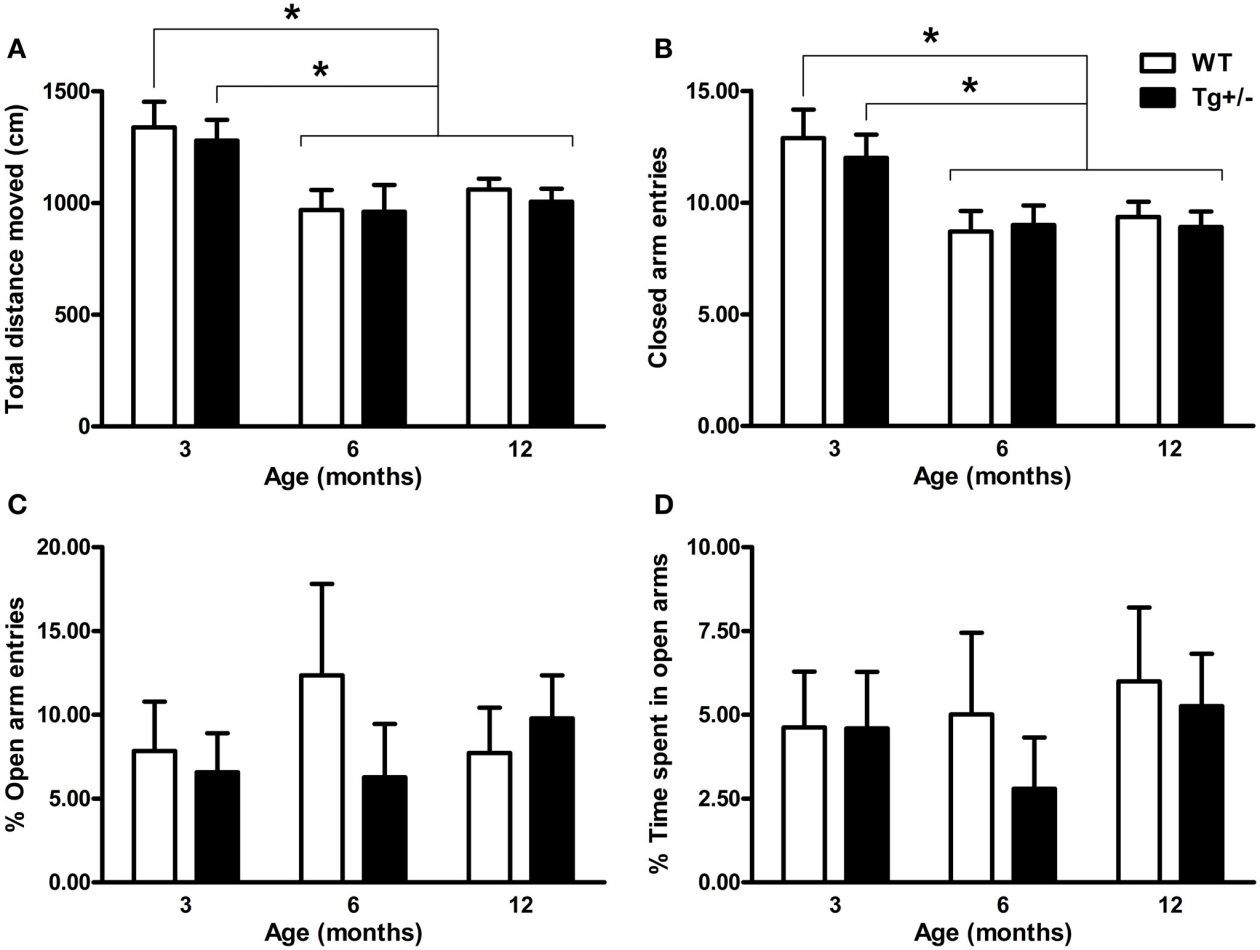
**Locomotor activity and anxiety-related behaviors of hemizygous transgenic McGill-R-Thy1-APP (Tg^+/−^) rats and wild-type (WT) littermates in the Elevated Plus Maze (EPM) test. (A)** Total distance moved. **(B)** Number of closed arm entries. **(C)** Percentage of open arm entries. **(D)** Percentage of time spent in open arms. Locomotor activity declined with age, regardless of the genotype **(A,B)**. No differences in anxiety-related behaviors were observed between Tg^+/−^ and WT rats at any age **(C,D)**. The number of animals tested was as follows: 9 WT and 10 Tg^+/−^ at 3 months, 7 WT and 9 Tg^+/−^ at 6 months, and 11 WT and 11 Tg^+/−^ at 12 months. Values are shown as the mean + s.e.m. ^*^p ≤ 0.05.

Two-Way ANOVA tests revealed significant main effects of age for “total distance moved” and “number of closed arm entries” [*F*_(2, 51)_ = 8.36, *p* = 0.001; *F*_(2, 51)_ = 9.29, *p* < 0.001, respectively]. By contrast, neither the main effects of genotype nor the age × genotype interactions were significant (*F* < 1 for all cases). These results suggest that locomotor activity changed with age, regardless of the genotype. *Post-hoc* analysis confirmed that both 6- and 12-month-old WT and Tg^+/−^ rats moved less distance and had a lower number of entries into closed arms than 3-month-old groups (*p* < 0.05 for all comparisons; see Figures 3A,B). Regarding “percentage of open arm entries” and “percentage of time spent in open arms,” neither the main effects of age nor the main effects of genotype nor the age × genotype interactions were significant (*F* < 1 for all cases; see Figures 3C,D), indicating that levels of anxiety were similar among all experimental groups.

### Open field test

**Figure 4:**
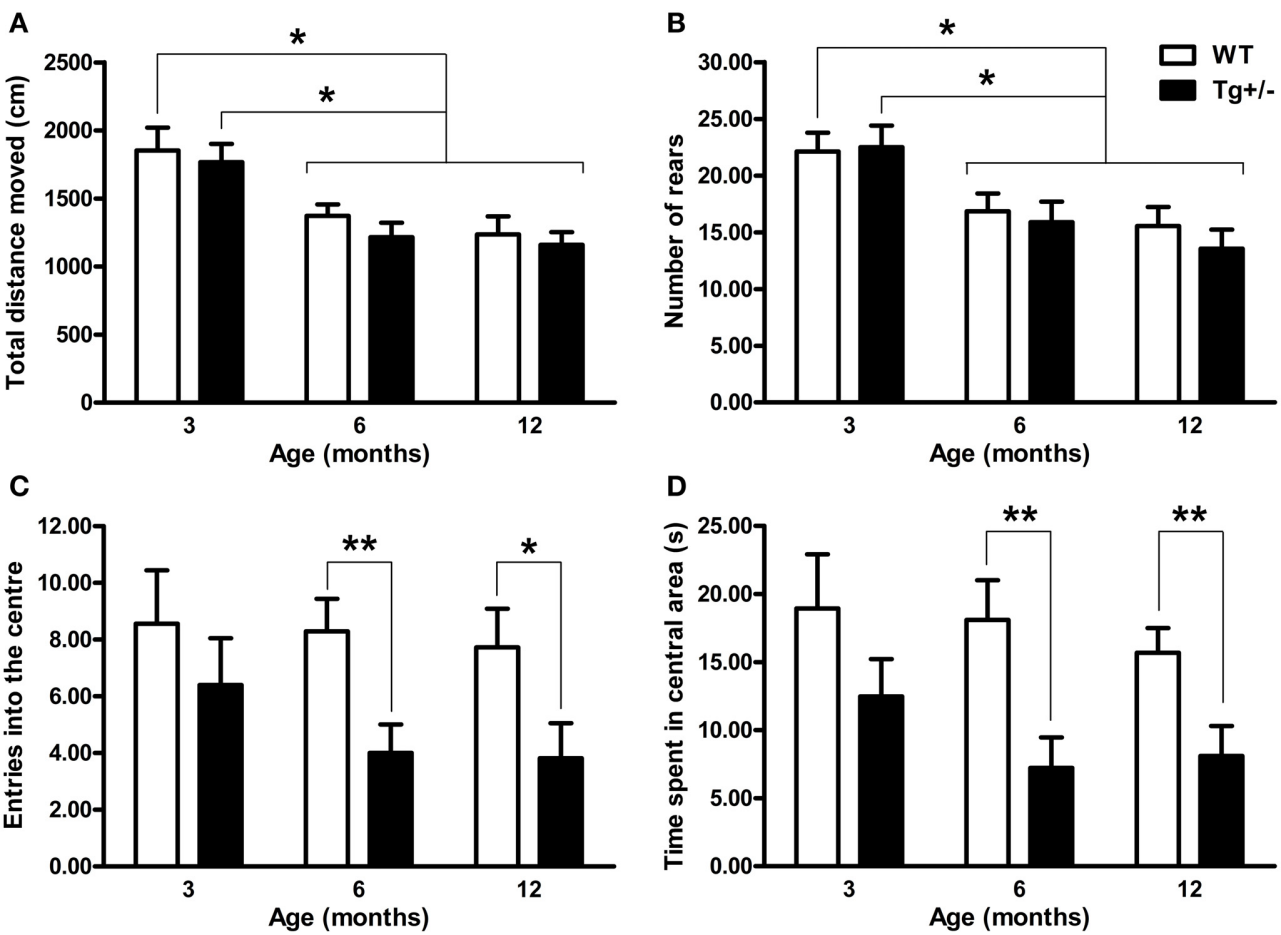
**Locomotor activity, exploration and anxiety-related behaviors of hemizygous transgenic McGill-R-Thy1-APP (Tg^+/−^) rats and wild-type (WT) littermates in the Open Field (OF) test. (A)** Total distance moved. **(B)** Number of rears. **(C)** Entries into the center. **(D)** Time spent in the central area. Regardless of the genotype, 6 and 12 months old rats showed less locomotion and rearing activity than 3 months old rats **(A,B)**. Tg^+/−^ rats of 6 and 12 months of age showed increased anxiety with a lower number of entries into center **(C)** and less time spent in the central area **(D)** than WT littermates. The number of animals tested was as follows: 9 WT and 10 Tg^+/−^ at 3 months, 7 WT and 9 Tg^+/−^ at 6 months, and 11 WT and 11 Tg^+/−^ at 12 months. Values are shown as the mean + s.e.m. ^*^p ≤ 0.05; ^**^p ≤ 0.01.

When “total distance moved” and “number of rears” were analyzed by Two-Way ANOVA tests, the main effects of age were significant [*F*_(2, 51)_ = 13.98, *p* < 0.001; *F*_(2, 51)_ = 11.10, *p* < 0.001, respectively] but neither the main effects of genotype nor the age × genotype interactions were significant (*F* ≤ 1 for all cases). *Post-hoc* multiple comparisons confirmed that locomotor and rearing activity declined with age, since 6- and 12-month-old WT and Tg^+/−^ rats moved less distance and presented a lower rearing frequency than 3-month-old groups (*p* < 0.05 for all comparisons; see Figures 4A,B). Relative to anxiety levels, the Two-Way ANOVA tests for “number of entries into the center” and “time spent in central area” indicated that neither the main effects of age nor the age × genotype interactions were significant (*F* ≤ 1 for all cases). However, the main effects of genotype showed significant differences [*F*_(2, 51)_ = 8.46, *p* = 0.005; *F*_(2, 51)_ = 13.87, *p* < 0.001, respectively]. *Post-hoc* analyses revealed that 6- and 12-month-old Tg^+/−^ rats showed a lower number of entries and spent less time in the center of the OF than WT groups (*p* < 0.05 for all comparisons; see Figures 4C,D). In contrast, 3-month-old Tg^+/−^ rats did not differ from their WT littermates in none of the variables analyzed (see Figures 4C,D). Since the interactions age × genotype did not reach statistical significance, the lack of differences between 3 month-old WT and Tg^+/−^ groups should be taken with caution (Tybout et al., 2001).

### Novel object recognition test

**Figure 5:**
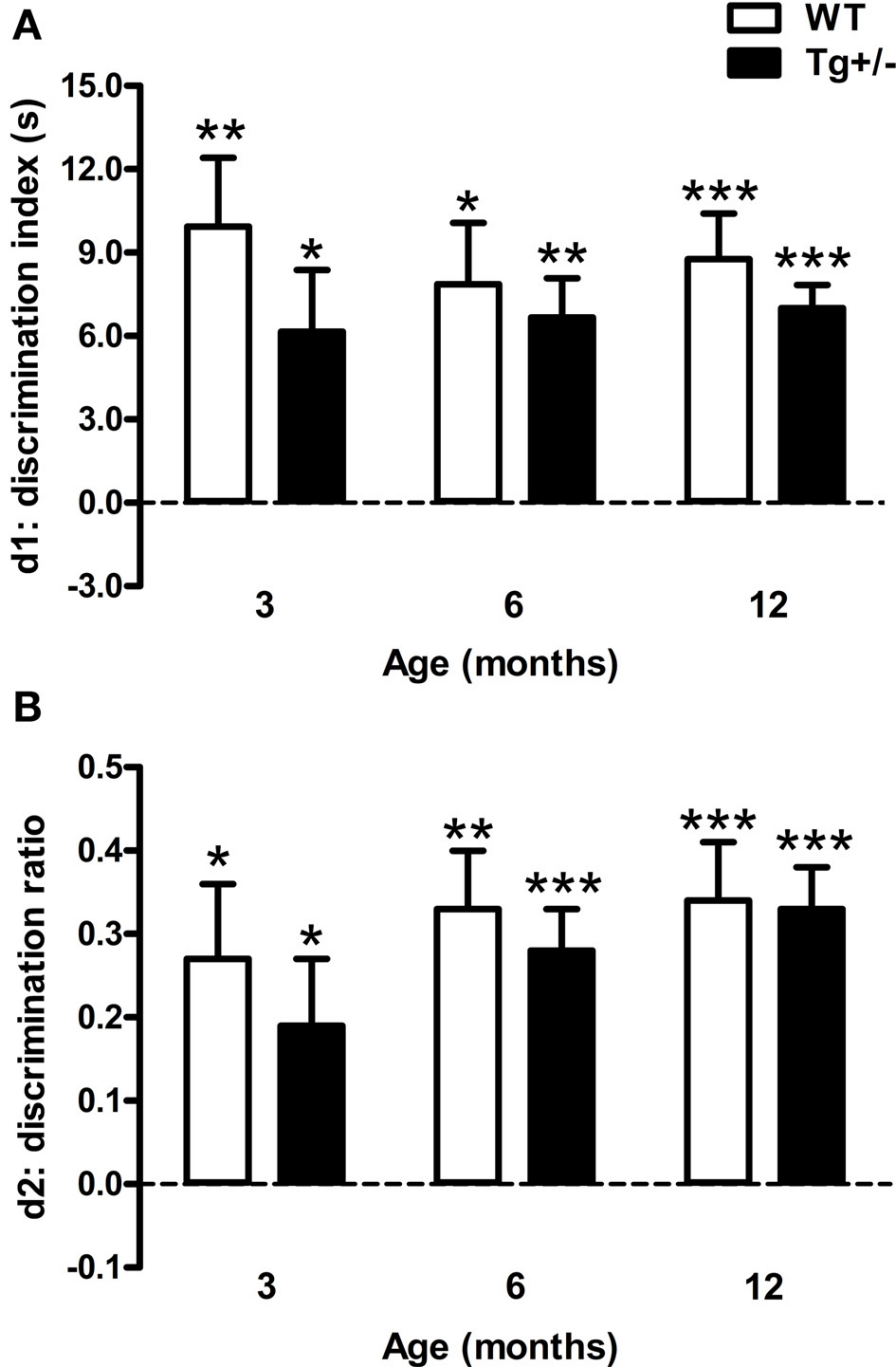
**Performance of hemizygous transgenic McGill-R-Thy1-APP (Tg^+/−^) rats and wild-type (WT) littermates in the Novel Object Recognition Test (NORT). (A)** Discrimination index (d1). **(B)** Discrimination ratio (d2). At all the ages tested, both genotypes recognized the novel object. The dashed lines indicate an equivalent exploration time for the two objects (chance level). The number of animals tested was as follows: 9 WT and 10 Tg^+/−^ at 3 months, 7 WT and 9 Tg^+/−^ at 6 months, and 11 WT and 11 Tg^+/−^ at 12 months. Values are shown as the mean + s.e.m. ^*^p ≤ 0.05; ^**^p ≤ 0.01; ^***^p ≤ 0.001 vs. chance (dashed lines).

The discrimination index (d1) and discrimination ratio (d2) were not statistically different between genotypes or ages [Two-Way ANOVA tests. d1. Genotype: *F*_(1, 51)_ = 2.25, *p* = n.s.; Age: *F*_(2, 51)_ < 1; Genotype × age: *F*_(2, 51)_ < 1. d2. Genotype: *F*_(1, 51)_ < 1; Age: *F*_(2, 51)_ = 1.25, *p* = n.s.; Genotype × age: *F*_(2, 51)_ < 1]. One sample *t*-tests indicated that d1 and d2 scores were significantly different from those expected by chance for all experimental groups (*p* < 0.05 for all cases; see Figures 5A,B). These results demonstrate that WT and Tg^+/−^ rats were able to recognize the novel object from the familiar one at all ages tested.

### Y-maze test

**Figure 6:**
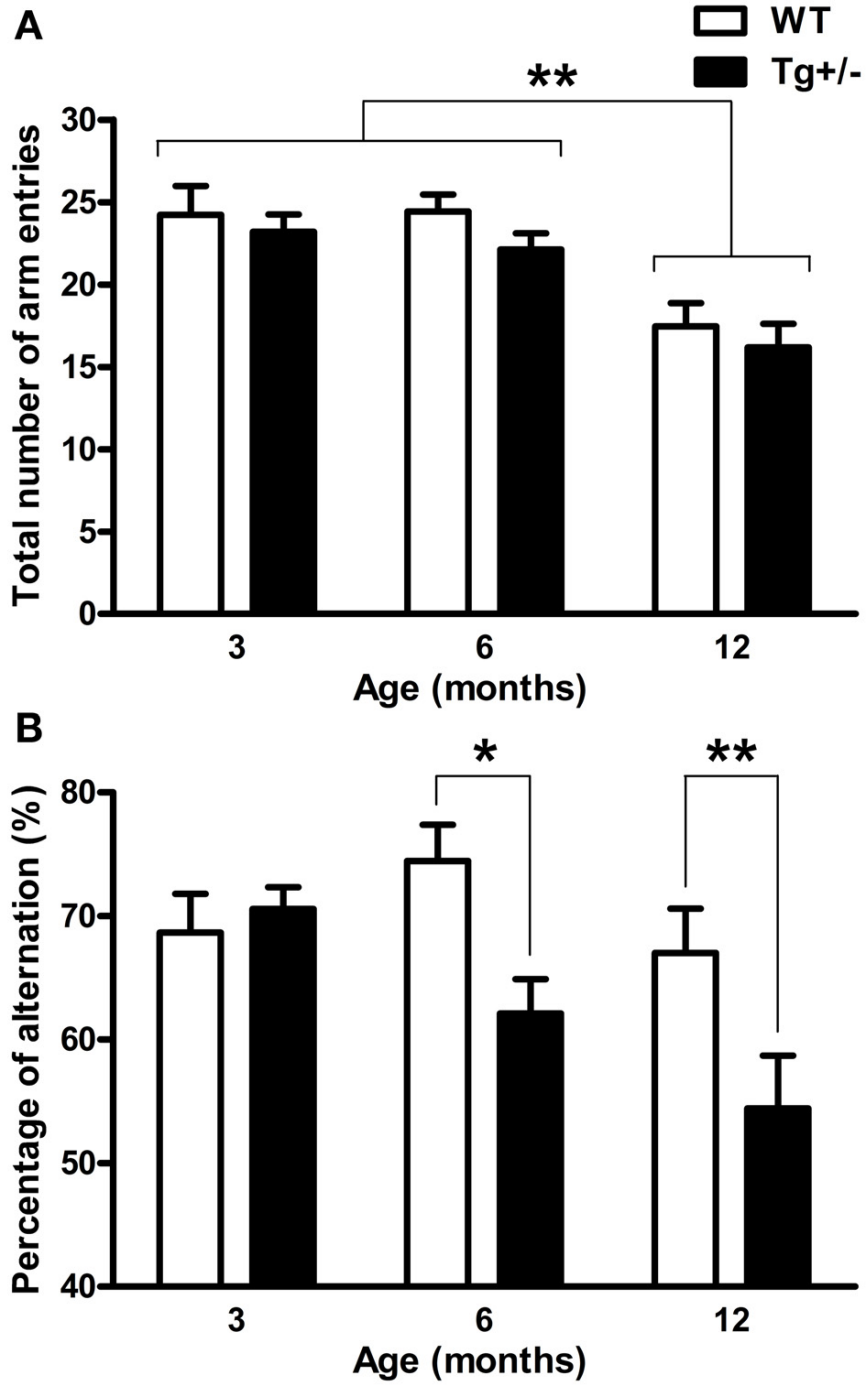
**Spontaneous alternation in the Y-maze test. (A)** Total number of arm entries. **(B)** Percentage of alternation. Regardless of the genotype, 12 months old rats showed less total number of arm entries than 3 and 6 months old rats **(A)**. At 6 and 12 months of age, hemizygous transgenic McGill-R-Thy1-APP (Tg^+/−^) rats exhibited an impairment in spontaneous alternation compared with wild-type (WT) groups **(B)**. The number of animals tested was as follows: 9 WT and 10 Tg^+/−^ at 3 months, 7 WT and 9 Tg^+/−^ at 6 months, and 11 WT and 11 Tg^+/−^ at 12 months. Values are shown as the mean + s.e.m. ^*^p ≤ 0.05; ^**^p ≤ 0.01.

A significant main effect of age for “total number of arm entries” [*F*_(2, 51)_ = 17.52, *p* < 0.001] was revealed by a Two-Way ANOVA test. Neither the main effect of genotype nor the age × genotype interaction were significant (*F* < 1 for both cases). Regardless of the genotype, *post-hoc* analysis showed that 12-month-old WT and Tg^+/−^ rats displayed a lower number of arm entries than 3- and 6-month-old groups (*p* < 0.01 for all comparisons; see Figure 6A). When the “percentage of alternation” was also analyzed by a Two-Way ANOVA test, the main effects of age and genotype and the interaction were all significant [*F*_(2, 51)_ = 4.48, *p* = 0.016; *F*_(2, 51)_ = 7.81, *p* = 0.007; *F*_(2, 51)_ = 3.13, *p* = 0.05, respectively]. *Post-hoc* tests indicated that 6- and 12-month-old Tg^+/−^ rats showed a lower percentage of alternation than their WT littermates (*p* = 0.020 and *p* = 0.006, respectively; see Figure 6B), while 3-month-old groups displayed similar levels of alternation (see Figure 6B). These results indicate that Tg^+/−^ rats displayed a spontaneous alternation impairment at 6 and 12 months of age.

### Morris water maze test

#### Cued learning

**Figure 7:**
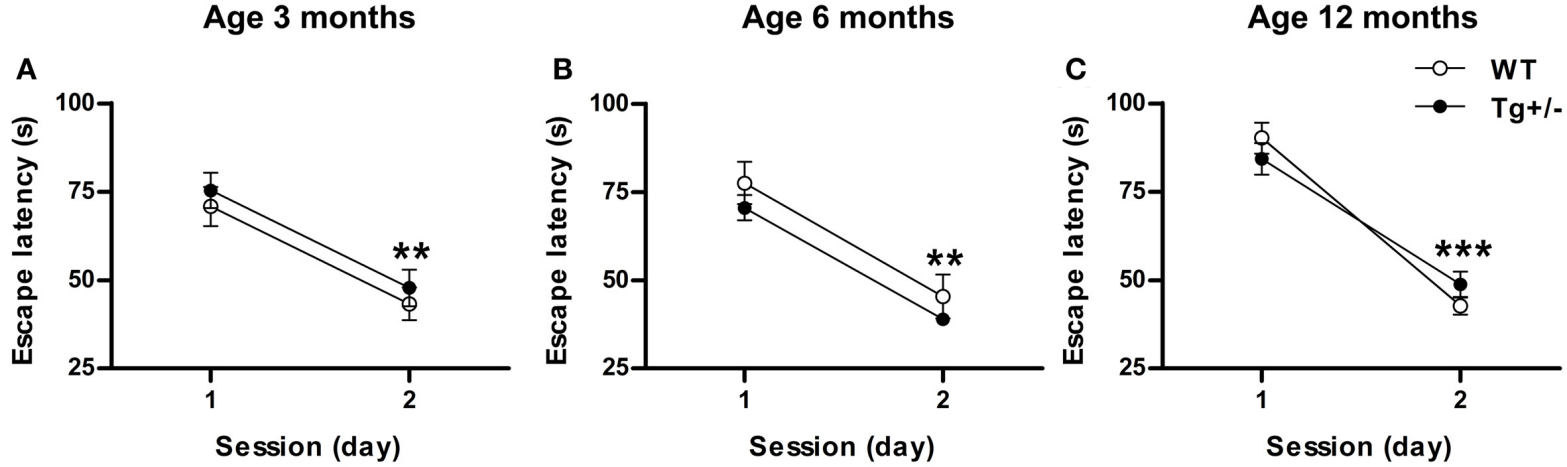
**Hemizygous transgenic McGill-R-Thy1-APP (Tg^+/−^) rats showed an unimpaired cued learning in the Morris Water Maze (MWM) test**. Escape latency of wild-type (WT) and Tg^+/−^ groups at 3 **(A)**, 6 **(B)** and 12 **(C)** months of age. At all ages tested, both genotypes reduced their escape latencies to similar levels from day 1 to day 2. The number of animals tested was as follows: 9 WT and 10 Tg^+/−^ at 3 months, 7 WT and 9 Tg^+/−^ at 6 months, and 11 WT and 11 Tg^+/−^ at 12 months. Values are shown as the mean ± s.e.m. ^**^p ≤ 0.01; ^***^p ≤ 0.001 vs. day 1.

At the three ages studied, *post-hoc* multiple comparisons showed that, during the second day, both genotypes significantly reduced their latency to reach the visible platform to similar levels (*p* < 0.01 for all cases; see Figures 7A–C) [Two-Way mixed ANOVA test. 3 months old. Day: *F*_(1, 17)_ = 66.57, *p* < 0.001; Genotype: *F*_(1, 17)_ < 1; Day × genotype: *F*_(1, 17)_ < 1. 6 months old. Day: *F*_(1, 14)_ = 145.87, *p* < 0.001; Genotype: *F*_(1, 14)_ = 1.44, *p* = n.s.; Day × genotype: *F*_(1, 14)_ < 1. 12 months old. Day: *F*_(1, 20)_ = 400.14, *p* < 0.001; Genotype: *F*_(1, 20)_ < 1; Day × genotype: *F*_(1, 20)_ = 8.27, *p* < 0.01]. These results suggest that transgenic groups were not visually impaired and showed no deficit in swimming ability.

#### Spatial learning

**Figure 8:**
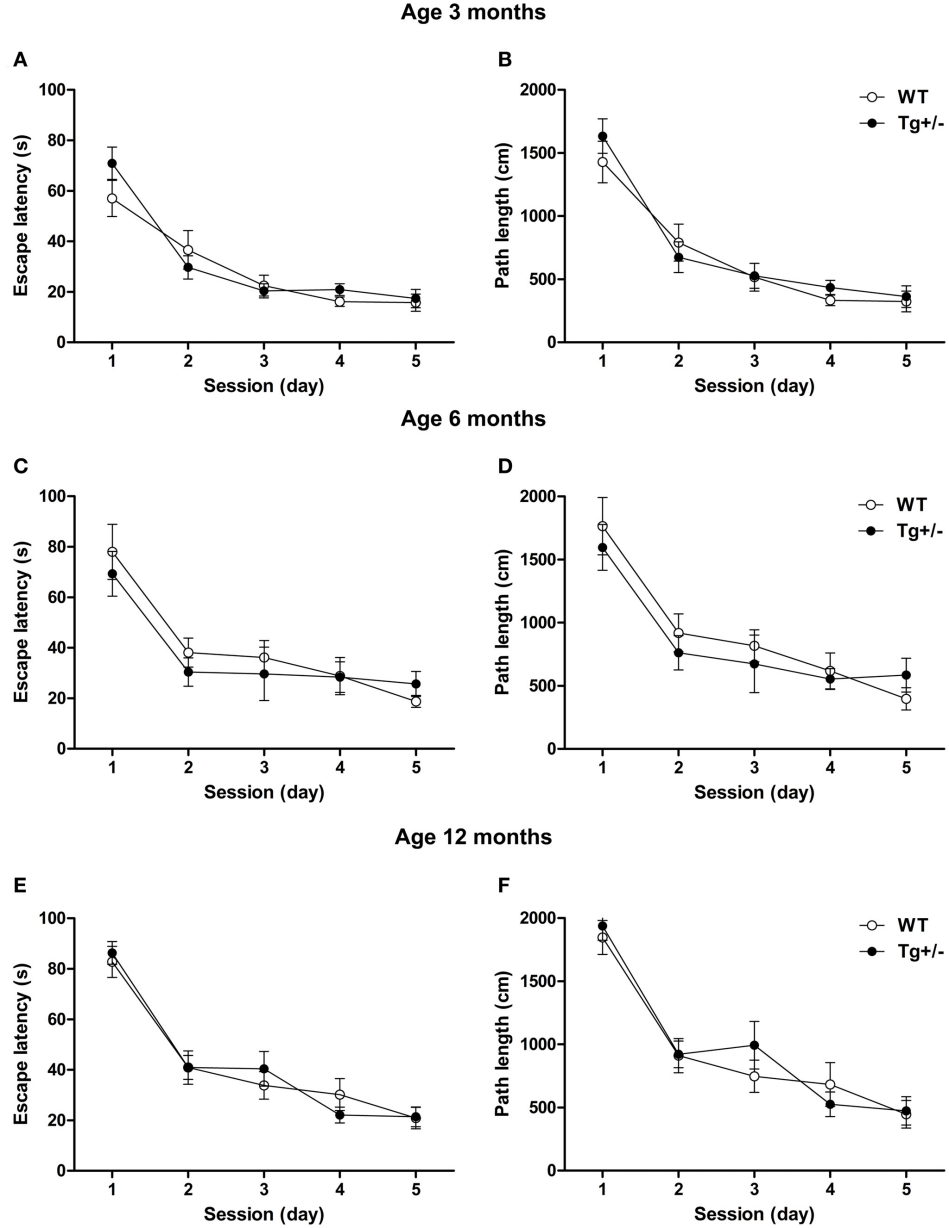
**Spatial learning of hemizygous transgenic McGill-R-Thy1-APP (Tg^+/−^) rats and wild-type (WT) littermates in the Morris Water Maze (MWM) test**. Escape latency and path length of Tg^+/−^ and WT rats at 3 **(A,B)**, 6 **(C,D)** and 12 **(E,F)** months of age. At all ages tested, both genotypes reduced their escape latencies and paths length at an equivalent rate across days. The number of animals tested was as follows: 9 WT and 10 Tg^+/−^ at 3 months, 7 WT and 9 Tg^+/−^ at 6 months, and 11 WT and 11 Tg^+/−^ at 12 months. Values are shown as the mean ± s.e.m.

Two-Way mixed ANOVA tests, with day as within-subject factor and genotype as between-subject factor, indicated that 3, 6, and 12 months old rats significantly decreased their escape latencies and distances swum to reach the hidden platform across days, regardless of the genotype [Escape Latency. 3 months old. Day: *F*_(4, 68)_ = 40.36, *p* < 0.001; Genotype: *F*_(1, 17)_ < 1; Day × genotype: *F*_(4, 68)_ = 1.6, *p* = n.s. 6 months old. Day: *F*_(4, 56)_ = 20.42, *p* < 0.001; Genotype: *F*_(1, 14)_ < 1; Day × genotype: *F*_(4, 56)_ < 1. 12 months old. Day: *F*_(4, 80)_ = 61.36, *p* < 0.001; Genotype: *F*_(1, 20)_ < 1; Day × genotype: *F*_(4, 80)_ < 1. Distance swum. 3 months old. Day: *F*_(4, 68)_ = 48.24, *p* < 0.001; Genotype: *F*_(1, 17)_ < 1; Day × genotype: *F*_(4, 68)_ < 1. 6 months old. Day: *F*_(4, 56)_ = 21.14, *p* < 0.001; Genotype: *F*_(1, 14)_ < 1; Day × genotype: *F*_(4, 56)_ < 1. 12 months old. Day: *F*_(4, 80)_ = 50.67, *p* < 0.001; Genotype: *F*_(1, 20)_ < 1; Day × genotype: *F*_(4, 80)_ < 1]. *Post-hoc* analyses confirmed that, at any age, escape latencies and distances swum did not differ between WT and Tg^+/−^ groups on any day of the learning phase (*p* = n.s. for all comparisons), indicating that all experimental groups were able to learn the task at an equivalent rate (see Figures 8A–F). Moreover, both genotypes displayed similar swimming speeds at all ages (see Figure 1 in Supplementary Material), suggesting similar levels of motivation to solve the task.

#### Spatial reference memory

**Figure 9:**
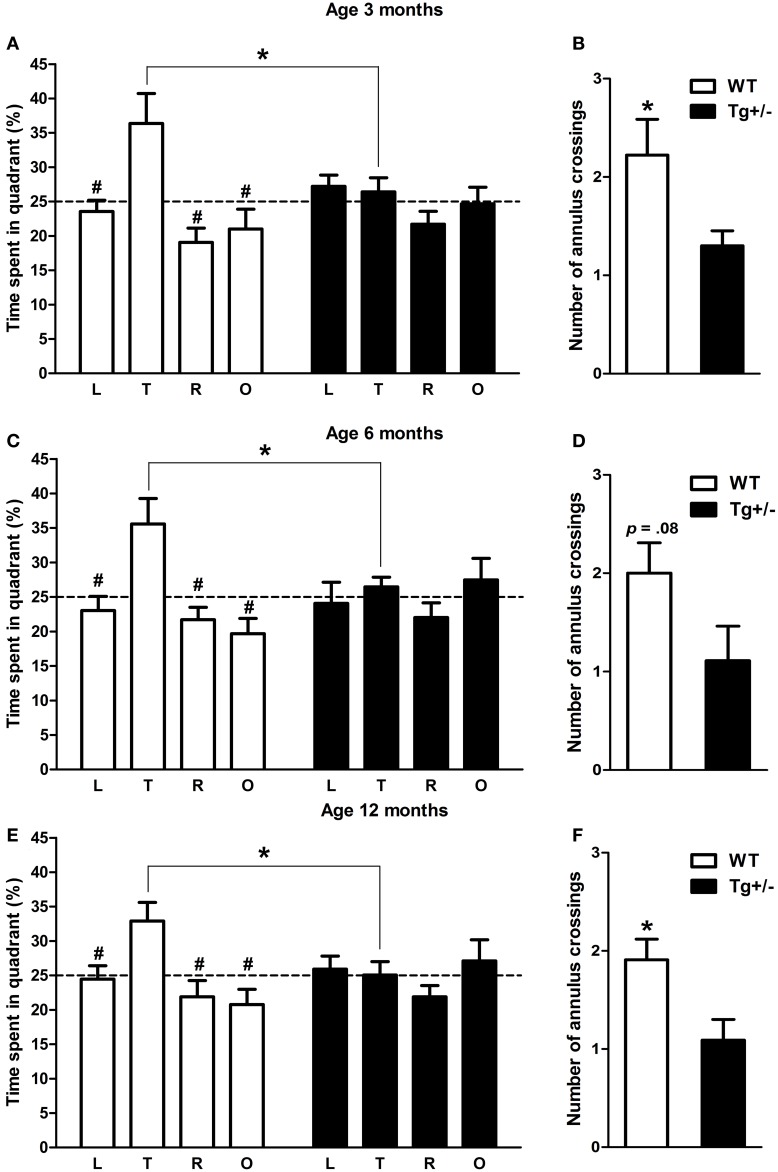
**Spatial reference memory impairment in hemizygous transgenic McGill-R-Thy1-APP (Tg^+/−^)**. Percentage of time spent in each quadrant of the Morris water maze and number of annulus crossings during the probe trial at 3 **(A,B)**, 6 **(C,D)** and 12 **(E,F)** months of age. At the three ages tested, WT groups spent significantly more time in the quadrant where the platform was previously located (target quadrant) **(A,C,D)**. By contrast, Tg^+/−^ groups spent similar amount of time in each one of the four quadrants **(A,C,D)**. Furthermore, Tg^+/−^ groups crossed the annulus zone significantly less times than WT groups (except at 6 months of age when a strong tendency was detected; **B,D,F**), indicating a spatial reference memory impairment. Dashed lines indicate the percentage of time spent in each one of the quadrant predicted by chance (25%). L, left quadrant; T, target quadrant; R, right quadrant; O, opposite quadrant. The number of animals tested was as follows: 9 WT and 10 Tg^+/−^ at 3 months, 7 WT and 9 Tg^+/−^ at 6 months, and 11 WT and 11 Tg^+/−^ at 12 months. Values are shown as the mean ± s.e.m. ^#^*p* ≤ 0.05 vs. percentage of time spent in target quadrant; ^*^p ≤ 0.05.

Two-Way mixed ANOVA tests, with quadrant as within-subject factor and genotype as between-subject factor, were performed to analyze the amount of time that experimental groups spent in each one of the quadrant of the MWM when platform was removed. At the three ages analyzed, results showed that the main effects of quadrant and the quadrant × genotype interactions were significant, in contrast to the main effects of genotype [3 months old. Quadrant: *F*_(3, 51)_ = 5.49, *p* = 0.02; Genotype: *F*_(1, 17)_ < 1; Quadrant x genotype: *F*_(3, 51)_ = 2.73, *p* = 0.04. 6 months old. Quadrant: *F*_(3, 42)_ = 3.74, *p* = 0.02; Genotype: *F*_(1, 14)_ < 1; Quadrant × genotype: *F*_(3, 42)_ = 2.70, *p* = 0.05. 12 months old. Quadrant: *F*_(3, 60)_ = 2.87, *p* = 0.04; Genotype: *F*_(1, 20)_ < 1; Quadrant × genotype: *F*_(3, 60)_ = 2.74, *p* = 0.04]. *Post-hoc* tests revealed that 3, 6, and 12 months old WT rats spent significantly more time in the quadrant where the platform was located (target quadrant) than in the remaining quadrants (*p* < 0.05 for all comparisons; see Figures 9A,C,E). On the contrary, Tg^+/−^ rats spent similar amount of time in each one of the quadrant at any age (*p* = n.s. for all comparisons; see Figures 9A,C,E). Furthermore, Student's *t*-tests revealed that 3 and 12 months old WT rats crossed the annulus zone significantly more time than Tg^+/−^ rats (*p* < 0.05 for both cases; see Figures 9B,F). A strong tendency was also detected for the 6 months old WT group (see Figure 9D). Overall, these results indicate that Tg^+/−^ rats had a significant impairment to remember the location of the platform as compared to WT animals. Regarding swimming speed and thigmotactic swimming, no differences were detected in probe trials (see Figures 1, 2 in Supplementary Material).

## Discussion

Hemizygous McGill-R-Thy1-APP transgenic rats represent an interesting model of early AD pathology as the presence of Aβ is limited to the intracellular compartment. While the longitudinal accumulation of Aβ in its homozygous counterpart has been recently documented (Iulita et al., 2014), little is known regarding the progression of the Aβ intracellular accumulation in the heterozygous counterpart and of the behavioral outcomes. In order to address this issue, we investigated the occurrence of human Aβ40 peptides after 2% SDS solubilization in hippocampal homogenates from 3-, 6-, and 12-month-old Tg^+/−^ rats by using an ELISA specific for human Aβ peptide. We were able to detect accumulation of Aβ40 immunoreactive material which remained at similar levels from 3 to 12 months of age. This finding is in agreement with the data reported in homozygous McGill-R-Thy1-APP rats (Iulita et al., 2014). The impossibility to detect Aβ42 peptide may be explained by the low amount of this peptide in the 2% SDS homogenates and the dynamic range of the ELISA test used in this experiment (between 1.56 and 100 pg/ml). Yet, the presence of SDS-resistant Aβ oligomers was detected by Western blot in 6- and 12-month-old animals. Since an amino-terminal anti-Aβ was used it was not possible to determine if these oligomers were composed of Aβ40, Aβ42 or both. To complement the structural and neurochemical characterization, we performed a longitudinal behavioral analysis of male hemizygous transgenic McGill-R-Thy1-APP (Tg^+/−^) rats, in tightly controlled conditions. To avoid high variability already reported in Tg^+/−^ groups (Leon et al., 2010) we followed 2 strategies: (1) exclusion of females rats, which are known to introduce confounding factors in behavioral tests (Mehta et al., 2013) and (2) the evaluation of at least 9 animals in each Tg^+/−^ group to increase statistical power. As it was mentioned in the “Materials and Methods” Section, the groups of animals that underwent the behavioral assessment were not the same used to study Aβ accumulation.

### Emotional behavior

There are several reports on murine models of AD, aimed at understanding the impact of amyloid formation on learning and memory but few are focused on emotional behavior. In this study, we analyzed the effect of genetic modification of APP on EPM and OF tests in Tg^+/−^ rats in view of the tendency of AD patients to be either apathetic or agitated (Chung and Cummings, 2000; Senanarong et al., 2004). We were unable to detect differences in anxiety-related behaviors between Tg^+/−^ and WT rats in the EPM test in the present study. Unchanged anxiety levels assessed by EPM were described in transgenic mice lacking amyloid plaques (Moran et al., 1995; Lalonde et al., 2002; Lee et al., 2004; Boon et al., 2010) and in two models with Aβ plaques (Arendash et al., 2001; Le Cudennec et al., 2008; Blanchard et al., 2009). It is important to note that behavior in EPM was not assessed in any of the transgenic rat models of AD described so far (Do Carmo and Cuello, 2013) and that our results in Tg^+/−^ rats, lacking extracellular Aβ deposition even at late stages, are in agreement with the concept that changes of EPM behavior appear only in transgenic models of AD with an extensive neuropathological phenotype (Savonenko et al., 2003; Lee et al., 2006; Lalonde et al., 2012). It is important to note that the levels of exploration of open arms seem to be low as compared to other reports (see Figures 3C,D). This effect could be attributable to the levels of illumination used (80–90 lux in the open arms). To rule out the possibility of a floor effect that could be masking an anxiety phenotype in Tg^+/−^ rats in the EPM, future studies are needed to investigate the behavior of these animals in this test under different experimental conditions.

Only one transgenic rat model of AD (the Tg6590) has been previously characterized for its performance in the OF. This transgenic rat model showed increased Aβ precursor protein (APP), cerebrovascular deposits, few diffuse plaques and increased phosphorylated tau at 15 months of age. At 9 months old these animals showed no differences either in the number of entries into center or in the time spent in the central area of the OF, as compared to control group (Kloskowska et al., 2010). By contrast, here we show that Tg^+/−^ rats are more anxious in the OF test at 6 months of age. This behavior was also observed at 12 months of age, suggesting the robustness of this phenotype. It is worth noting that this anxious phenotype was observed in the OF test but not in the EPM test. Previously, it was reported that the OF and the EPM operationalize different aspects of emotional behavioral (Trullas and Skolnick, 1993; Ramos et al., 1998). This lack of inter-test correlations has been attributed to the multidimensional nature of emotional behavior (Ramos, 2008). Therefore, it could be possible that the higher anxiety levels detected in Tg^+/−^ rats in the present study, reflect an specific dimension of emotional behavior linked to OF activity. Finally, it is important to note that both tests (EPM and OF) were sensitive to the age-related decline in spontaneous locomotor activity and exploration (see Figures 3A,B, 4A,B).

In summary, even if the emergency (Holmes, 2001) or the light/dark box (Pinton et al., 2011) tests were not assessed in this study, our report is the first analysis of the anxiety disturbances in a transgenic rat model of early AD that does not develop Aβ plaques. It is of interest that the anxiety pattern described here for the Tg^+/−^ rats appear to closely mimic neuropsychiatric symptoms described in some pre-clinical AD patients.

### Cognitive behavior

Ideally, genetically modified animal models of AD not only have to replicate some of the hallmark neuropathology, such as plaque-like amyloid accumulations and tau deposition, but also should reproduce some of the cognitive impairments relevant to the disease. In this context, near all of the transgenic mice models with excessive Aβ either via transgenic overexpression of mutant hAPP and/or its processing enzymes, typically present static cognitive deficits that are evident early in life and progressive deficits that are age-related and associated with increased concentrations of deposited or unbound assemblies of Aβ (Kobayashi and Chen, 2005). By contrast to transgenic mice in which a wide battery of cognitive tests were performed, a narrower battery of tests were used in transgenic rat models of AD. These included MWM, Fear Conditioning, and Novel Object Recognition and Location (NORL) test (Leon et al., 2010; Iulita et al., 2014). Early description of the cognitive performance of hemizygous transgenic McGill-R-Thy1-APP rat in the MWM showed that at 3 months of age (*n* = 4) the learning curve was undistinguishable from that of WT (*n* = 8) rats while at 13 months of age, Tg^+/−^ rats (*n* = 4) exhibited a mild spatial learning deficit reflected by a significantly increased escape latency, compared to WT (*n* = 12), during the third day of the learning phase. Moreover, spatial reference memory, assessed during the probe trial, was not impaired at both ages, although a trend could be seen in the 3 month-old group (Leon et al., 2010). To further characterize the cognitive abilities of Tg^+/−^ rats, in this study we analyzed the performance of these animals in three different tasks including MWM, Novel Object Recognition (NOR) and Y-maze tests at 3, 6 and 12 months old. Using a larger number of Tg^+/−^ animals, we were able to detect, in the MWM, a spatial reference memory deficit early at 3 months of age, which persisted at 6 and 12 months. This deficit cannot be explained by a slower learning curve, as in contrast to the original report (Leon et al., 2010), we did not find in Tg^+/−^ rats any impairment in the learning phase of the MWM test. These discrepancies can be explained both by the number of animals used and by the gender of the animals utilized (males and females in the first report while males only in our study).

Memory tasks sensitive to hippocampal functions such as contextual fear conditioning (FC), NORL and Y-maze alternation have been described in different transgenic mice models of AD (Corcoran et al., 2002; Dineley et al., 2002; Ohno et al., 2004; Devi and Ohno, 2012; Mitani et al., 2013; Nagahara et al., 2013). However, none of these tests were assessed in transgenic rats until a recent report in which hemizygous transgenic McGill-R-Thy1-APP (Tg^+/−^) rats showed reduced auditory Fear Conditioning (FC) and reduced contextual and cued FC recall (Iulita et al., 2014). In the same study, Tg^+/−^ rats displayed an impairment in the Novel Object Recognition and Location (NORL) task (Iulita et al., 2014). The impairment described in the NORL test was not reproduced by Tg^+/−^ in the NOR task performed in our study. This could be due to the differential stringency of the tasks as five objects were used in the previous report (Iulita et al., 2014). Although both tests are hippocampal-dependent, the NORL is a more complex task that includes the spatial component that is absent in the classic NORT test, and may explain the discrepancy between results. While in the former test one unfamiliar object had to be discriminated among a total of five objects during the novel object recognition phase of the task, we employed a more classical approach with two dissimilar objects (one unfamiliar and one familiar) that had to be discriminated during the retention trial. Discrepancies in results reveal that when the complexity of the task is increased, recognition memory may be also impaired in this transgenic rat model.

Spontaneous alternation in the Y-maze test estimates the willingness to explore novel stimuli, or to avoid familiar stimuli, and is an appropriate paradigm to asses working memory and/or behavioral disinhibition, signs also manifest in AD. Also, the total number of arm entries in the Y-maze is a parameter used to assess sensoriomotor capacities. In this study, we did not find significant differences in the total number of arm entries between Tg^+/−^ and WT rats. However, a significant decline was detected in the percentage of alternation in Tg^+/−^ rats at 6 and 12 months old. We could not ascribe this reduced rate in spontaneous alternation to behavioral disinhibition since performance of Tg^+/−^ rats in locomotor and exploratory parameters in the EPM and OF did not differ from WT. Therefore, it is likely that reduced spontaneous alternation reflects spatial working memory impairment. Poor performance in Y-maze appears in young mice of several single transgenic AD models at 2–4 months, when there are no plaque-like deposits, but these mice have had high levels of soluble Aβ in their nervous systems from early postnatal life (Lalonde et al., 2012). These observations are in agreements with our results, since Tg^+/−^ rats lack parenchymal Aβ plaques but thus accumulate soluble oligomers intraneuronally.

## Concluding remarks

In conclusion, in the present time-course study, we investigated emotional and cognitive symptoms in Tg^+/−^ rats. The most relevant result is that transgenic rats showed, from adulthood (6-month-old) to middle age (12-month-old), increased anxiety levels and working memory impairment. Moreover, as early as 3 months of age, transgenic rats showed an impaired spatial reference memory in comparison to WT rats. This deficit was also observed at 6 and 12 months of age. At all ages, recognition memory and spatial learning were spared. Given that Tg^+/−^ rats capture many behavioral features of early stages of AD, this new model may represent an important tool for the neuroscience community to enable future studies in basic and translational AD research.

### Conflict of interest statement

The authors declare that the research was conducted in the absence of any commercial or financial relationships that could be construed as a potential conflict of interest.
