# Supplementary material for: Longitudinal analysis of the behavioral phenotype in a novel transgenic rat model of early stages of Alzheimer's disease
Source: Front Behav Neurosci. 2014 Sep 16;8:321. doi: 10.3389/fnbeh.2014.00321 (PMC4165352; doi:10.3389/fnbeh.2014.00321)
Supplement: Supplementary file 1 [file DataSheet1.DOCX]

***Supplementary Material***

**Longitudinal analysis of the behavioral phenotype in a novel transgenic rat model of early stages of Alzheimer’s disease**

**Pablo Galeano^1,2^, Pamela V. Martino Adami^1^, Sonia Do Carmo^3^, Eduardo Blanco^4^, Cecilia Rotondaro^1^, Francisco Capani^2^, Eduardo M. Castaño^1^, A. Claudio Cuello^3^, Laura Morelli^1^***

^1^Fundación Instituto Leloir, Instituto de Investigaciones Bioquímicas de Buenos Aires, CONICET, Ciudad Autónoma de Buenos Aires, Argentina

^2^Instituto de Investigaciones Cardiológicas “Prof. Dr. Alberto C. Taquini” (ININCA), Universidad de Buenos Aires and CONICET, Ciudad Autónoma de Buenos Aires, Argentina

^3^Department of Pharmacology and Therapeutics, McGill University, Montreal, QC, Canada

^4^Departament de Pedagogia i Psicologia, Facultatd'Educació, Psicologia i Treball Social, Universitat de Lleida, Lleida, Spain

*** Correspondence:** Dra. Laura Morelli, Laboratorio de Amiloidosis y Neurodegeneración, Fundación Instituto Leloir, IIBBA (CONICET), Av. Patricias Argentinas 435, C1405BWE, Ciudad Autónoma de Buenos Aires, Argentina, e-mail: [lmorelli@leloir.org.ar](mailto:lmorelli@leloir.org.ar)


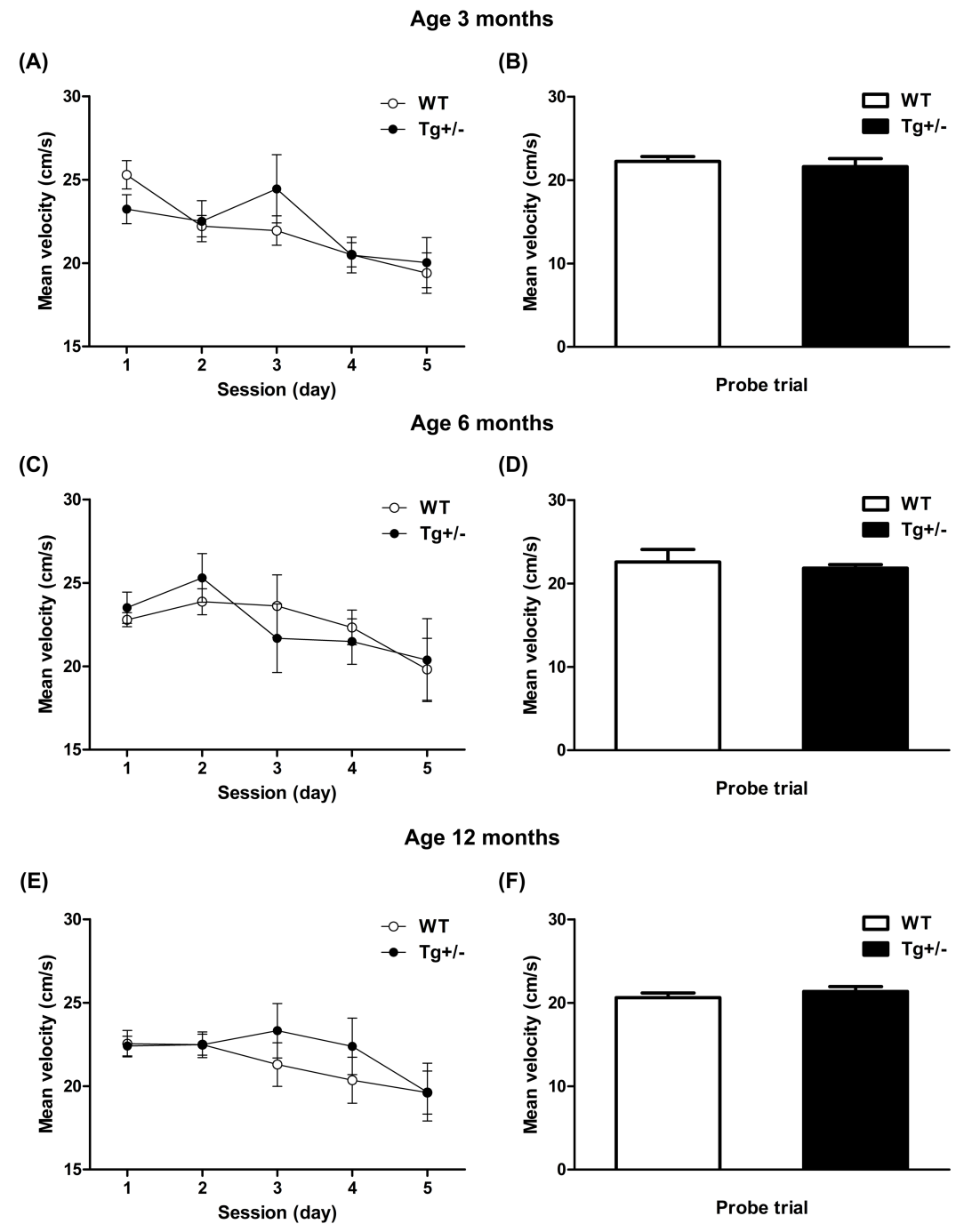


Supplementary Figure 1. Swimming speed during the learning phase and probe trial in the spatial reference memory version of the Morris Water Maze (MWM). (A) At 3 months of age, swimming speed decreased across the five days of the learning phase but it was not affected by genotype (Day: *F*_(4, 68)_ = 8.25, *p* < 0.001; Genotype: *F*_(1, 17)_ < 1; Day x genotype: *F*_(4, 68)_ = 1.54, *p* = n.s.). (C and E) At 6 and 12 months of age, swimming speed was affected neither by day nor by genotype (6 months old. Day: *F*_(4, 56)_ = 2.22, *p* = 0.08; Genotype: *F*_(1, 14)_ < 1; Day x genotype: *F*_(4, 56)_ < 1. 12 months old. Day: *F*_(4, 80)_ = 2,04, *p* = 0.09; Genotype: *F*_(1, 20)_ < 1; Day x genotype: *F*_(4, 80)_ < 1). (B, D and F) Student’s t-tests indicated that both genotypes showed similar swimming speeds during the probe trial at all ages tested (*p* = n.s. for the three t-tests). The number of animals tested was as follows: 9 WT and 10 Tg+/- at 3 months, 7 WT and 9 Tg+/- at 6 months, and 11 WT and 11 Tg+/- at 12 months. Values are shown as the mean ± SEM or as the mean + SEM.


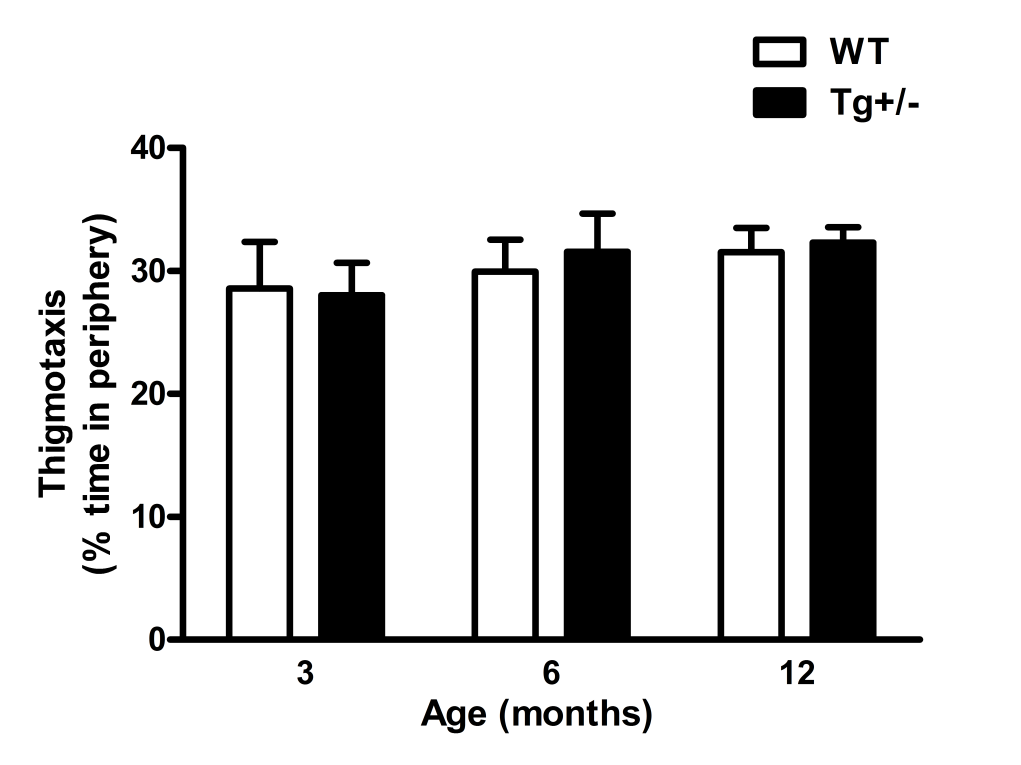


**Supplementary Figure 2. Thigmotactic swimming during probe trials in the spatial reference memory version of the Morris Water Maze (MWM).** Student’s t-tests showed that both genotypes spent similar amount of time in the outer ring zone during the probe trial, at the three ages tested (*p* = n.s. for all cases). These results support that anxiety did not affect the performance of the animals during the probe trials.The number of animals tested was as follows: 9 WT and 10 Tg+/- at 3 months, 7 WT and 9 Tg+/- at 6 months, and 11 WT and 11 Tg+/- at 12 months. Values are shown as the mean + SEM.
